# Supplementary material for: Are carbon emissions trading and green financial instruments synergistic? -Comprehensive quantitative research based on content analysis
Source: PLoS One. 2024 Mar 7;19(3):e0298601. doi: 10.1371/journal.pone.0298601 (PMC10919601; doi:10.1371/journal.pone.0298601)
Supplement: S1 Data — (ZIP) [file pone.0298601.s001.zip › Data/地级市Z_F工作报告.zip!/╣π╢1⁄2/╤⌠╜¡╩╨/└┤╫╘╣1⁄2╓┌║┼í╛┬φ┐╦╩2╛▌═°í┐_2019.docx]

各位代表：

　　现在，我代表市人民政府向大会作政府工作报告，请予审议，并请政协各位委员和其他列席人员提出意见。

　　一、2018年工作回顾

　　刚刚过去的一年，是极不平凡的一年。欣逢改革开放40周年、阳江建市30周年，年初，习近平总书记在参加十三届全国人大一次会议广东代表团审议时赋予广东“四个走在全国前列”的新使命，10月底，又亲临广东视察提出四个方面重要指示要求，对广东寄予殷切期望，为阳江发展指明方向。一年来，我们沿着总书记指引的道路奋勇前行，按照省委、省政府和市委的决策部署，坚持稳中求进工作总基调和高质量发展要求，深入实施“以海兴市、绿色发展”战略，以“项目落实年”为重要抓手，着力打基础、解难题、办大事，新兴主导产业集聚壮大，城市扩容提质加快推进，交通网络建设明显提速，“三大攻坚战”实现良好开局，经济社会发展取得新的成效。预计全市实现地区生产总值1350亿元，增长3.5%；地方一般公共预算收入62.6亿元，增长3.2%；规模以上工业增加值215亿元，增长3%；固定资产投资391亿元，增长4%；社会消费品零售总额750亿元，增长9.5%；城镇居民人均可支配收入29300元，增长6.3%，农村常住居民人均可支配收入16800元，增长9.5%。

　　（一）经济发展提质增效。

　　扎实推进“六稳”，全市经济运行总体平稳，质量效益逐步提升。固定资产投资逐步回暖，工业投资扭转上年持续负增长的局面，在一季度实现正增长，并连续7个月平均增长21.2%，其中装备制造业投资增长98.1%。工业企业效益继续向好，规模以上工业利润总额增长33%。地方财政收入结构持续优化，税收占比达71.8%。第17届刀博会成交金额突破23亿元、参展参会人数超过11万人次，分别比上届增长180%和30%，进一步提升了我市五金刀剪产业在国内行业中的领军地位。招商引资势头强劲，引进项目193个、投资总额827.9亿元，分别增长85.6%、79.2%，其中10亿元以上项目16个，增长45%。召开全市民营企业座谈会，设立中小微企业公共服务平台和信贷风险补偿资金，出台支持实体经济发展的若干政策措施，全面落实省、市“实体经济十条”，为企业减负超16亿元，民营经济增长贡献率达63%。

　　（二）主导产业实现突破。

　　合金材料产业集群初具规模，国内主要大型不锈钢生产企业先后落户，甬金冷轧、宏旺冷轧、新兴铸管二期等项目顺利动工，广青热轧项目加快建设，66家紧固件企业抱团进驻，成为国内最大的高端不锈钢生产集聚区之一。风电产业生态体系逐步形成，海上风电开发规模大、速度快、配套好，1000万千瓦海上风电项目全部完成核准，首期第一批4个风电项目推进顺利；

　　国内海上风电装备制造核心企业相继落户，粤水电等3个项目竣工投产，明阳整机项目加快建设，金风科技、中车电机、龙马铸件等3个项目正式动工，国家海上风电装备质检中心获准筹建，风电全产业链基地显现雏形。绿色食品产业加速集聚，“中国调味品之都”落户阳西，美味鲜等5个项目建成投产，卡夫·亨氏等10个项目加快建设，致美斋、广味源等13个项目签约落户，全部达产将实现年产调味品300万吨，成为国内最大的调味品生产基地。新能源产业发展壮大，阳江核电5号机组成功并网，阳江调峰储气库项目动工建设。特色旅游业提质发展，预计接待游客2337万人次，旅游总收入突破300亿元，分别增长12%和15%，悦榕庄、喜来登等一批国际知名酒店项目加快前期工作，“一部手机畅游阳江”平台试运行。现代农业加快发展，顺欣海洋渔业公司创建农业产业化国家重点龙头企业通过公示，新增11个省名牌农产品，闸坡、溪头等现代渔港建设扎实推进，首届农民丰收节成功举办。

　　（三）城市功能不断完善。推进城市向南向海，阳江滨海新区城南片区基本建成，投资140亿元的滨海中央商务区全面动工。新江南路、洛东大道、城南东路等项目竣工通车，站前广场和站前路建成开放，康泰北路等10条市政道路PPP项目开工建设，新区与市区的关键道路节点进一步打通。交通基础设施建设换挡提速，完成投资83亿元，接近“十二五”期间总投资，是历年来阳江交通投入最多的一年。江湛铁路顺利建成通车，阳江阔步迈进“高铁时代”。汕湛高速阳春段建成通车，沈海高速开阳扩建工程、G325线北惯至白沙段改线等18个项目完成年度建设任务，广东滨海旅游公路阳江段、沈海高速阳茂扩建工程、阳江港10万吨级进港航道改造等4个项目动工建设，东融西联大格局加快形成。漠阳湖公园、南山公园、森林公园一期、北湖公园改造等一批城市生态景观项目和9个社区体育公园对外开放，东汇城、盈信广场等商业综合体建成开业。园区基础设施建设加快推进，平整土地350万平方米，新建园区道路12公里、标准厂房13万平方米，启动建设高新区工业污水处理厂等一批环保设施。扎实开展城市综合管理考核和“六大”专项整治行动，城市面貌更加整洁靓丽。

　　（四）改革创新全面深化。市县机构改革稳步实施，市级新组建部门全部挂牌。政府系统关键领域40项改革任务、18项“微改革”工作事项和“自我革新”微改革活动扎实推进。“放管服”改革不断深化，取消证明事项93项，基本建成全市一体化在线政务服务平台和网上中介服务超市。在全省率先把税务票房和印章刻制点引入政务服务大厅，开办企业便利度跃升至全省第7位。创建国家高新区取得实质性进展，成功纳入国家科技部2018年专家咨询调研名单。新增41家高新技术企业，增长128%。重点实验室建设取得突破，高功率激光应用实验室挂牌成立，首家省级企业重点实验室获批。科技创新平台加快建设，新增2家院士工作站、2家省级新型研发机构和10家省级工程技术研究中心，规模以上工业企业研发机构覆盖率达25%。国家知识产权试点城市建设深入开展，专利申请量4000件、专利授权量2950件、新注册商标3168件，增长均超过20%。大力推进标准化战略，制定《陶瓷刀具的要求》，实现了我市国际标准零的突破。

　　（五）生态优势巩固提升。集中开展固体废物污染防治排查整治等4个专项大行动，扎实推进中央、省环保督察问题整改，办结案件135宗；深入开展“散乱污”企业“利剑”专项行动，限期整改企业352家，完成整改266家，一批环保突出问题得到基本解决。全面落实河长制，开展江河湖库“五清”和“清四乱”等专项行动，扎实推进漠阳江流域综合整治，连围河整治工程、五旦水闸重建工程竣工，解决了一批困扰多年的河湖管理难题。阳东区、阳春市和阳西县垃圾处理设施升级改造基本完成，市区5个片区总长150公里的截污及排水管网改造工程陆续动工，建成后将有效解决市区污水直排、雨污分流不彻底问题。27个乡镇饮用水水源保护区全部完成规范化建设，4个县级以上集中式饮用水水源保护区水质达标率100%。全面铺开国家森林城市创建工作，开展林业四大重点生态工程，造林更新面积9.14万亩，闸坡镇被认定为省级森林小镇。

　　（六）民生福祉持续增进。投入民生资金167.1亿元，占一般公共预算支出74.5%。省、市十件民生实事基本完成。城镇新增就业3.68万人，城镇登记失业率控制在2.41%。教育工作取得突破性进展，全部县（市、区）获得“广东省推进教育现代化先进县（市、区）”称号，10所义务教育学校启动建设，资助学生7.8万人，发放补助资金6567.6万元。卫生健康事业加快发展，市人民医院新住院大楼投入使用，市中医院二期工程动工建设，4家三级公立医院启动医联体建设，5家县办公立医院、2家中心卫生院升级建设进展顺利，医疗“强基层”和“互联网+医疗”取得积极成效。文化体育建设扎实推进，建成44个行政村（社区）综合性文化服务中心示范点，体育健儿在亚洲帆板锦标赛、第15届省运会分别获金牌5枚、9枚，创历史最好成绩。底线民生保障水平稳步提升，失能特困人员治疗与护理实现全覆盖，累计分配公租房4758套，改造农村危房3333户。乡村振兴取得阶段性成效，88个省定贫困村基本完成村庄基础设施项目建设，6748个自然村完成“三清理三拆除三整治”工作，占总数的83.4%；累计7.1万相对贫困人口实现预脱贫。防灾减灾救灾能力不断提高，成功防御强台风“山竹”和罕见洪水，实现重大灾情无重大伤亡、无重大险情的防控目标。食品安全监管取得新成绩，成为粤东西北地区首个省食品安全示范城市创建试点。积极防范化解金融、房地产等领域重大风险，解决了一批房地产项目历史遗留问题。加强安全生产监督管理，有效遏制各类违法犯罪，扫黑除恶专项斗争取得阶段性成效，社会治安形势持续好转，社会大局保持和谐稳定。

　　过去的一年，市政府落实全面从严治党要求，以党建新成效促进政务水平新提升。认真执行市人大及其常委会决定，自觉接受市政协监督，办理省、市人大代表建议97件，省、市政协委员建议案和提案257件，办复率均为100%。加强法治政府建设，配合市人大出台我市首部地方性实体法规——《阳江市漠阳江流域水质保护条例》。深入开展作风建设，激发干部干事创业的精气神，营造了良好的发展氛围。

　　与此同时，双拥共建、民族宗教、外事侨务、对台、人防、地震、供销、档案、地方志、妇女儿童、残疾人等工作取得新成绩。

　　各位代表！过去的一年，成绩来之不易。这是习近平新时代中国特色社会主义思想科学指引的结果，是党中央、国务院和省委、省政府及市委运筹帷幄、正确领导的结果，是市人大、市政协大力支持、有效监督的结果，是珠海市等有关方面对口帮扶、携手共进的结果，更是全市人民共同努力、不懈奋斗的结果。在此，我代表市人民政府，向全市广大干部群众，向人大代表和政协委员，向各民主党派、工商联、无党派人士，向驻阳江部队、武警官兵、中央和省驻阳江单位，向离退休老同志，向关心、支持阳江改革发展的社会各界人士，向多年帮扶阳江发展的珠海市及有关单位，表示衷心的感谢！

　　在肯定成绩的同时，我们也清醒地认识到，经济社会发展还存在一些问题和短板，主要表现在：一是经济增速有所回落。受经济调结构、增质量和加快“僵尸企业”出清等因素叠加影响，我市地区生产总值、规模以上工业增加值、固定资产投资增速回落，外贸进出口增长未能扭负为正，与预期目标存在差距。二是项目推进不快。由于征地拆迁、资金筹措、前期工作等方面原因，部分项目未能如期开工、竣工，影响有效投资增长和企业产能及时释放。三是城乡发展不平衡。我市城镇化、工业化进程加快，但农村发展不充分问题突出，基础设施较为滞后，农村产业发展形式单一，农村经济传统发展模式和发展路径亟待破解。四是环保形势不容乐观。空气和水环境质量呈下降趋势，排污管网建设等历史欠账较多，工地扬尘管理、固体废物监管不到位，企业环保主体意识不强，环境违法行为时有发生。五是教育、医疗等民生领域还有不少短板。优质资源供给不足、分布不均等问题依然存在，教育教学质量仍需提高，公共卫生服务能力和重点专科建设仍需加强。六是干部队伍建设有待提升。一些干部业务不熟、能力不强、不敢担当，不想为、慢作为现象依然存在，与新时代新任务新要求不相适应。对此，我们将认真研究，竭力解决，绝不辜负群众的信任和期望！

　　二、2019年工作安排

　　今年是新中国成立70周年，是全面建成小康社会关键之年，也是全面贯彻落实习近平总书记视察广东重要讲话精神，按照省委李希书记对阳江提出的要求打造沿海经济带重要战略支点、宜居宜业宜游现代化滨海城市关键之年，做好全年工作意义重大。从全国、全省的发展大局来看，我国发展仍处于重要战略机遇期，改革开放再出发汇聚磅礴伟力，粤港澳大湾区建设、“一核一带一区”区域发展新格局全面推进，我们迎来了千载难逢的重大机遇。我市位于粤港澳大湾区、北部湾城市群和海南自贸区交汇处，经过多年的艰苦奋斗，发展基础更加坚实，发展动力更加充足，后发优势更加明显，在全省大局中的地位更加凸显。站在新的历史起点上，我们要始终保持战略定力，以永不懈怠的精神状态和一往无前的奋斗姿态，全面贯彻落实打造沿海经济带的重要战略支点、宜居宜业宜游的现代化滨海城市的行动方案，奋力开创阳江发展新局面。

　　今年市政府工作的总体要求是：高举习近平新时代中国特色社会主义思想伟大旗帜，全面贯彻党的十九大和十九届二中、三中全会以及中央经济工作会议精神，全面贯彻落实习近平总书记对广东重要讲话和对广东工作一系列重要指示精神，学习贯彻落实省委十二届六次全会及市委七届八次全会精神，统筹推进“五位一体”总体布局，协调推进“四个全面”战略布局，坚持稳中求进工作总基调，坚持新发展理念，坚持推动高质量发展，坚持以供给侧结构性改革为主线，坚持深化市场化改革、扩大高水平开放，积极主动融入粤港澳大湾区，深入落实省委“1+1+9”工作部署，统筹推进稳增长、促改革、调结构、惠民生、防风险、保稳定各项工作，进一步做好“六稳”，提振市场信心，打好“三大攻坚战”，着力提高发展平衡性和协调性，保持经济持续健康发展和社会大局稳定，为全面建成小康社会收官打下决定性基础，奋力打造沿海经济带重要战略支点、宜居宜业宜游的现代化滨海城市，为广东实现“四个走在全国前列”、当好“两个重要窗口”作出阳江贡献。

　　今年经济社会发展的预期目标：生产总值增长6-6.5%；规模以上工业增加值增长8.5%以上；固定资产投资增长9%左右；社会消费品零售总额增长8.5%；外贸进出口总额增长2%；一般公共预算收入增长6.5%；居民人均可支配收入增长与经济增长基本同步；消费价格涨幅控制在3%以内；城镇登记失业率控制在3%以内；每万元生产总值能耗完成省下达年度任务。

　　为实现上述年度目标，重点做好八个方面的工作：

　　（一）努力促进经济运行稳中向优

　　着力扩大有效投资。充分发挥政府投资的引导作用，全力推动年度投资268亿元的112个市重点项目建设，确保22个项目建成投产、40个项目全面动工。规范有序推行PPP模式，加快推动已招入社会资本方的总投资156亿元的19个PPP项目建设。紧跟国家产业政策和投资导向，高起点谋划一批重大项目。加快投融资体制改革，落实激发民间投资活力政策措施，积极扩大民间投资。

　　充分促进居民消费。大力发展假日经济和会展经济，积极培育定制消费、体验消费等消费新热点，多渠道多形式扩大消费领域和消费群体。逐步缩小城乡居民消费差距，加快发展农村电子商务，建设20家乡村商贸驿站。建立健全消费后评价制度和消费领域信用体系建设，维护消费者合法权益，创造安全放心的消费环境，加快形成强大市场。

　　努力稳定对外贸易。制定落实促进外贸稳增长政策措施，优化外贸发展环境。办好第18届刀博会，组织参加中国国际进口博览会等30场展会，积极帮助企业开拓市场。深化国际贸易“单一窗口”国标版推广工作，提高通关效率，降低通关成本。坚持进口与出口并重，多举措扩大先进设备、原材料进口贸易。大力培育跨境电子商务、市场采购和外贸综合服务企业等新业态，积极挖掘外贸新增长点。

　　大力发展民营经济。全面落实省“实体经济新十条”“民营经济十条”等政策，修订完善我市扶持政策，清理和精简涉企收费，化解融资难、融资贵问题，全面降低企业成本。强化暖企惠企行动，办好市中小微企业公共平台和鼍企在线，深入开展企业集中服务月活动，建立完善社会信用和市场监管体系，进一步优化营商环境。抓好“个转企、小升规”工作，大力培育行业龙头骨干企业，新增年产值100亿元以上企业1家、50亿元以上企业1家、20亿元以上企业4家、10亿元以上企业4家。

　　（二）做大做强主导产业

　　快速壮大两大新兴产业。围绕打造千亿级合金材料产业集群，全力推动广青热轧在1月底、宏旺冷轧在6月、甬金冷轧在10月建成投产；大力发展不锈钢制品精加工，推动开宝不锈钢在7月、永贤不锈钢在8月建成投产；加快推进新型建材（特种钢）及铸管铸件产业园建设，大力发展紧固件等下游产业。围绕建设世界级风电产业基地，加快发展海上风电装备制造产业集群，推动明阳整机项目3月投产，力促金风科技、中车电机、龙马铸件等3个项目年内建成投产，推动国家海上风电装备质检中心、东方海缆等9个项目年内动工。

　　提升优化四大传统产业。落实新的工业企业技术改造三年行动计划，推动传统产业加快转型升级。扶持遴选出的5家五金刀剪龙头企业，以点带面推动五金刀剪产业高质量发展。推动近海浅水区首期第一、二批300万千瓦海上风电项目加快建设，开展近海深水区700万千瓦海上风电项目前期工作，加快阳江抽水蓄能电站主体工程、阳江调峰储气库建设，确保阳江核电6号机组9月投入商运，全力打造国家新能源基地。推进卡夫·亨氏、美味源等4个项目建成投产，力促致美斋、广味源等9个项目开工建设，加快发展调味品产业和农海产品精深加工，做大做优做强食品加工业。规划建设服装鞋帽产业园区，加强自主品牌建设和技术创新，振兴服装鞋帽产业。

　　力促招商引资新突破。持续保持招商引资良好势头，全年新签约项目100个以上、总投资400亿元以上，其中超10亿元项目8个以上。重点引进不锈钢餐厨用品、门窗制造等龙头企业以及合金材料其他分类企业，不断完善合金材料从基础原料到各类制品的完整产业链条。对标国际先进风电制造安装运维母港，推动顶尖风电企业以及海上风电研发中心、运维中心、大数据中心签约落户。依托卡夫·亨氏、厨邦等企业进行补链招商、精准招商，加速食品加工产业集聚发展。落实企业落户优惠政策，提高项目履约率、动工率和竣工率。

　　掀起园区提质建设大会战。按照推进高质量发展高新区现场会的部署，以园区道路、标准厂房、生活配套建设作为着力点，统筹推进全市产业园区64个基础设施（含配套）项目建设，加快完善产业园区各项配套，为企业落地提供更好环境。重点推进高新区建设，推动港口生活配套区工程和一批园区道路、标准厂房建设，把高新区打造成为全市新兴产业的聚集地、高质量发展的强大引擎。推动共建产业园开发建设，科学谋划园区扩园工作，适度增加园区容量。加大阳江港开发力度。实施公司化专业化管理模式，由市交投集团全面承担阳江港“开发、建设、运营、管理、服务”职能，适时引进战略投资者，加快阳江港东西两翼联结通道建设，推进10万吨级进港航道改造、11个5万吨级以上码头泊位建设，提高公共码头比例，促进港口码头泊位大型化、专业化发展，实现阳江港大规划、大建设、大发展。

　　因地制宜发展园区经济。珠海(阳江)产业转移工业园重点发展合金材料及制品、先进制造业、新能源新材料产业、大宗商品仓储物流产业。珠海(阳江万象)产业转移工业园重点发展五金机械、五金刀剪制造等产业。中山火炬(阳西)产业转移工业园重点发展绿色食品、新型建材等相关产业。阳春产业转移工业园重点发展钢铁、原材料、基础件和医药等产业。江城银岭产业园重点发展五金制品、金属制品、食品及传统服装等产业。滨海新区重点发展现代服务业。海陵试验区重点发展旅游业。其它工业园区立足自身基础和优势，努力推动特色产业集聚发展。

　　提高科技创新和金融服务能力。推动高新区成功创建国家高新区，中山火炬(阳西)产业转移工业园、阳春产业转移工业园成功创建省级高新区。培育具有较强竞争力的创新型企业，新增高新技术企业26家。加强创新平台建设，做大做强高新技术企业孵化中心，推动高功率激光应用实验室取得实质性进展，注重合金材料的研发和科研成果转换，新增1家省级企业重点实验室，新增省、市级工程技术研究中心20家以上。谋划建设公共技术服务平台，让中小企业共享工业设计、产品工艺、智能化改造、检验检测、生产物流等共性需求，有效降低企业发展成本。加强知识产权保护，推进中国阳江（五金刀剪）知识产权快速维权中心建设，实现专利申请量突破4500件。以高层次创新型人才为重点，加强创新人才队伍建设。完善科技金融服务体系，推进省科技金融综合服务中心阳江分中心建设。引进新的金融机构，加大金融服务实体经济力度。

　　（三）加快建设宜居滨海城市

　　加强城市规划引领。高起点谋划城市建设，融入生态景观和地域文化特色，全力打造现代化滨海城市。全面衔接粤港澳大湾区、北部湾、沿海经济带等上位规划，对接周边城市规划，推动区域统筹融合发展。逐步建立“多规合一”的规划体系，年内实现市区控规全覆盖。集中力量规划建设城南片区和滨海新城，重点围绕深茂铁路阳江站中央商务区建设，将城南组团打造成以总部经济、商贸中心、高端住宅为主的新城市中心；启动广湛高铁阳江站TOD规划建设；以对岸渔港为切入点，科学适度推进金朗岛开发建设；以特色文旅项目、广东滨海旅游公路建设等为抓手，突出滨海新城旅游主题。阳东区加快市区一体化进程，增强中心城区辐射带动能力。阳春市突出生态保护，致力打造市域北部宜商宜居宜游生态副中心。阳西县突出产城融合，以大规划推动产城大发展。海陵试验区围绕打造国际知名旅游生态海岛，全面铺开全域旅游建设。

　　推进基础设施建设大提速。编制《阳江市综合交通运输体系中长期发展规划》，构筑“东进、西联、成网、立体”的现代综合交通运输体系，打造粤港澳大湾区与粤西、北部湾城市群乃至大西南联结的重要交通枢纽。建成怀阳高速海陵岛大桥、G325线北惯至白沙段改建等5个项目，加快沈海高速阳江段扩建工程、广东滨海旅游公路阳江段等26个项目建设，力促广湛高铁阳江段、西部沿海高速阳江南联络线、阳江港大桥、海陵岛轨道交通等项目年内开工建设，推进阳江机场、海陵水上机场等项目前期工作，规划建设连接高铁站、机场和各组团之间的快速交通网络。完善阳阳铁路的集疏运功能，并向港口和工业区延伸。统筹规划建设市区市政道路，建成金平路一期，推进雅白线、阳东江台路南延线、中洲大道南延线等“三横四纵”城市快速路网项目建设，逐步完善中心城区道路设施。突出推进江海堤围加固达标、中型水闸重建、四围涝区整治，动工建设大河水库引水工程等漠阳江流域综合整治规划首批年度项目。

　　塑造城市发展新品质。大力创建国家森林城市，全面实施六大主题行动，推动“两江四岸”北岸湿地公园、共青湖公园、森林公园二期、滨海生态公园、儿童公园等项目加快建设，努力让群众乐享“家门口就在公园旁”的健康生活，全面提升城市生态宜居水平。加快完善城市功能配套，推动市档案馆、方志馆、党史馆（三馆合一）等项目建设。加强重点街区设计，打造标志性精品建筑。推进城市更新改造，启动滨海新区旧村庄等改造项目，注重保护修缮历史文化建筑，促进传统与现代融合发展，让城市留下记忆，让人们记住乡愁。

　　实施精细化城市管理。对标国内先进城市，注重运用法规制度标准管理城市，构建权责明晰、服务为先、管理规范的城市管理体系。强化城市综合管理考核，推动市容“六乱”整治常态化，重点整治城市管理“老大难”问题。大力提升城市美化水平，加强东风路等重点道路及周围建筑的优化改造，推进城南片区等重点区域和市区标志性建筑亮化工程，实施主要街道园林绿化增彩工程，让阳江这座城市更宜居、更具魅力。

　　（四）扎实推进乡村振兴战略

　　加快推进美丽乡村建设。推进农村人居生态环境综合整治，完成“三清理三拆除三整治”，60%以上村庄达到干净整洁村标准。完成阳东区、阳西县2个省级新农村示范片建设，推进“四好农村路”示范县（区）创建工作。加强镇村同治，建设美丽镇区。推动漠阳农耕文化遗产、革命遗址的传承保护与适度利用，加快建设岗美镇潭簕村和塘口镇横山村2个省定红色村。积极引导各类人才“上山下乡”，推动乡村人才振兴。

　　大力发展富民兴村产业。实施农业特色产业“一一三三”工程，建成6个以上“一村一品、一镇一业”农业特色专业镇、39个以上农业特色专业村，着力增加优质绿色农产品供给。积极建设现代农业产业园，各县（市、区）建设1个以上省级现代农业产业园。引导农村养殖散户转产转业，培育农业龙头企业、农民专业合作社、家庭农场等新型经营主体，新增省级品牌农产品5个以上、“三品一标”产品5个以上。发展林下经济，建设国家级示范基地1个、省级示范基地4个。

　　坚决打赢脱贫攻坚战。深化精准施策，实现累计7.6万相对贫困人口预脱贫。加快推进项目建设，完成省定贫困村创建新农村示范村考核目标任务。深入推进产业扶贫，大力发展优势特色扶贫产业，建设一批农业特色主导产业扶贫村。推动农村低保制度与扶贫开发政策有效衔接，加快补齐农村基础设施短板，完成932户贫困户危房改造。深入开展扶志教育活动，激发贫困群众内生动力。建立促进贫困户稳定脱贫和防范返贫的长效机制，夯实长远发展基础。

　　（五）推动改革开放再出发

　　深入推进重点领域改革。深化“放管服”改革，推进“一门式一网式”政府服务模式改革和审批制度改革，做到“四减一提速”。加快智慧城市建设，推动“粤省事”阳江专版上线，逐步完善政务服务网，打造“数字+”平台，实现政务服务水平提升和市民生活质量改善。全面完成市县机构改革，调整优化事业单位设置，扎实推进市级预算编制执行监督管理改革，以及市县财政事权和支出责任划分改革。完善国有资产管理体制，健全国有资产监督体系。加快经济体制改革，促进经济高质量发展。

　　主动融入粤港澳大湾区。在交通、产业、城镇、体制机制等方面深度对接融入，谋求在高快速铁路、产业集中集聚发展、重大平台建设等方面突破，接受粤港澳大湾区辐射带动。深化与港澳广深等核心城市的合作，吸引大湾区资源要素流入，加快流动性开放向制度型开放转变。发挥粤港澳大湾区辐射粤西重要战略支点作用，当好海南自贸区和北部湾城市群对接粤港澳大湾区的门户枢纽。

　　全面加强区域合作交流。积极对接中央、省的区域协调发展政策，加强北部湾城市群、泛珠区域、高铁沿线经济带合作，推进产业融合、资源共享、旅游联盟。主动对接珠海对口帮扶，突出产业招商、园区建设、社会民生和精准扶贫，推动不少于20个超亿元项目动工建设。完善企业“走出去”服务体系，拓展与欧美以及“一带一路”沿线国家和地区的经贸合作。

　　（六）着力促进文化旅游繁荣发展

　　提升城市文明程度。大力培育和践行社会主义核心价值观，深化中国梦主题宣传教育。扎实开展全国文明城市创建工作，争取2020年获得创建国家文明城市提名资格。加强公民道德建设，开展“阳江好人”推荐评选、“厚德善行讲堂”巡回宣讲等活动。普及文明礼仪规范，推进移风易俗，文明村镇覆盖率达80%。培育壮大志愿者队伍，注册志愿者达到20万人。

　　建设文化名城。以建设广东省水下文化遗产保护中心为契机，统筹优化广东海上丝绸之路博物馆片区规划，谋划建设广东海上丝绸之路旅游文化产业创意园，完善周边配套，开发相关文化旅游产业，擦亮“南海I号”世界级文化名片。改造利用旧船厂、旧糖厂等历史建筑，探索建设创意文化产业园。加强风筝、楹联、漆艺、阳江山歌等特色文化的传承和创新发展，谋划培育新兴文化业态和文化品牌。推动文体产业深度融合，办好帆船帆板比赛、马拉松赛和漠阳风筝文化节等活动，开发更多具有本土文化内涵、现代竞技精神的文体项目和产品。

　　完善公共文化服务体系。启动文化艺术中心等重大文化设施谋划工作，谋划建设名人纪念馆、名人故居，推进江城区、阳东区、阳西县博物馆建设，加快10个行政村（社区）综合性文化服务中心省级示范点建设；推进市体育综合训练馆建设和县（市、区）文化场馆升级改造、体育场馆建设，推动“三馆一站”和公共体育场馆逐步对外免费开放，增加优质公共文化服务供给，丰富人民群众文体生活。

　　擦亮全域旅游品牌。落实发展全域旅游工作海陵现场会部署，加快编制市县全域旅游规划，将旅游业培育成为支柱产业和幸福产业，打造国内著名滨海旅游目的地。坚持海陆联动，以海陵试验区创建国家全域旅游示范区为龙头带动，推动悦榕庄、喜来登等国际酒店品牌重点项目动工建设，积极争取省级重大滨海旅游产业项目落户，抓好闸坡、东平、沙扒、春湾等镇创建旅游风情小镇工作，支持春湾风景区等一批旅游景区提档升级。加快培育旅游新业态新产品，大力发展乡村旅游、森林旅游和特色民宿，推进旅游业与健康养老、生态休闲等深度融合。推广“粤菜师傅工程”和“旅游后备箱工程”，培育推介我市特色美食、优质土特产和优质旅游商品。深入开展旅游环境整治提升大行动，进一步优化全市旅游环境。

　　（七）大力加强生态文明建设

　　抓好环保督察反馈问题整改。巩固污染防治排查整治专项行动成果，持续抓好固体废物处理处置、黑臭水体整治、禽畜养殖污染治理、饮用水水源保护区违法违规建筑清拆等重点难点问题整改，力促整改工作落实。加强对中央、省环保督察案件的督导办理，完成余下22宗案件的整改销案。实行飞行检查制度，对正在推进和已经完成的整改任务强化跟踪督导检查，确保整改工作全面完成。

　　打好污染防治攻坚战。全面摸清全市污染源底数，深入实施打好污染防治攻坚战三年行动计划，全力守护天蓝地绿水清的家园。坚决打赢蓝天保卫战，重点加强城市扬尘治理，建立健全区域及城市间联防联控联治机制。聚力打好碧水保卫战，推动河长制湖长制全面见效，实施“让漠阳大地河更美”大行动，启动建设“万里碧道”阳江示范工程，高质量高标准推进500公里中小河流治理（二期）工程，加强入河入海排污口监管；加快污水处理设施建设，推动市区5个片区截污及排水管网改造工程建设取得实质性进展，城南、城北污水处理厂扩容提标工程年内建成，阳东区、阳春市整县推进村镇污水处理设施建设全面完成，阳西县整县推进村镇污水处理设施建设全面开展。扎实推进净土保卫战，深入开展土壤质量调查，抓好省级农村生活垃圾收运处理验收复检工作，推动固体废物处理处置重点工程建设。

　　构建绿色发展长效机制。完善部门环保责任清单，增强县镇基层环保执法力量。大力发展循环经济，推动高新区、中山火炬(阳西)产业转移工业园、阳春产业转移工业园开展园区循环化改造示范试点，加快先进节能环保技术、工艺和装备的研发和应用。严控高耗能、高污染项目准入，推广节能新技术、新产品。推广装配式建筑，城填新增绿色建筑面积35.5万平方米。推行绿色生活方式，加快新能源汽车推广应用，引导市民绿色出行。

　　（八）切实提高人民生活水平

　　办好人民满意的教育。优先发展教育事业，巩固拓展教育现代化成果。用三年时间实施基础教育再提升工程，使阳江的基础教育质量跃上新台阶。年内建成10所义务教育学校，新建3所义务教育学校和2所公办幼儿园，改扩建学校8所，新增义务教育学位2.5万个。实施“强师工程”，加快推进市县级教师发展中心建设，培养38名“阳江名师”。深化中小学教师“县管校聘”管理改革，促进县（市、区）域内教师均衡配置。建立完善教育教学质量抽测和监测体系，提升教育教学质量。结合本地新兴产业发展特点，大力发展职业教育。加强平安校园建设，提高校园安保水平。

　　加快健康阳江建设。开展市人民医院升级改造项目、市中医院二期建设，谋划新建1家二级医院。加强内科、外科等47个重点专科建设，每家三级综合性医院打造不少于1个省级高水平临床重点专科。实施“名医工程”，培养学科带头人，推动提升全市医生队伍整体素质。加大基层医疗卫生人才培养力度，开展66名产科、儿科医生转岗培训和209名全科医生培训。完善分级诊疗制度，县域内基层医疗卫生机构诊疗量占总诊疗量65%以上。建设危重病人救治中心，市县两级院前急救体系年底前建成投入使用。发展中医药特色服务，组建市中医“治未病”技术指导中心和“治未病”专科联盟。

　　完善社会保障体系。把稳就业摆在突出位置，城镇新增就业3万人，就业困难人员实现再就业2000人。持续提高低保、特困供养、残疾人“两项补贴”和孤儿基本生活保障标准，加大社会保险扩面征缴力度，完善全民医保体系。推进养老服务体系建设和老龄、慈善事业发展，强化农村留守儿童和困境儿童服务保障。坚持房子是用来住的、不是用来炒的定位，完善住房市场体系和住房保障体系，同时切实解决房地产项目历史遗留问题。

　　构建共建共治共享社会治理格局。推动扫黑除恶专项斗争向纵深发展，切实加强行业治理，提升黑恶犯罪打击效能，铲除黑恶势力滋生的土壤。深化平安阳江建设，严厉打击各类违法犯罪活动。坚持和发展“枫桥经验”，统筹推进各级公共法律服务中心建设，推动社会治理重心向基层下移。积极培育发展社会组织，新增社会组织200个。落实“七五”普法规划，做好司法行政工作。打好防范化解金融风险攻坚战，完成阳江农商行等3家农合机构改制，推动阳西农商行开业，大力防范网贷风险，守住不发生系统性金融风险的底线。坚守安全生产、食品药品安全等工作底线，推进大应急平台建设，提升防灾减灾救灾水平。扎实做好双拥共建工作，巩固军民团结。

　　各位代表！人民群众对美好生活的向往就是我们的奋斗目标。新的一年，我们将突出提升劳动者就业水平、全域推进农村人居环境整治等方面，投资49.2亿元，办好十件民生实事，大力发展民生事业，努力补齐全面小康短板，加快全面建成小康社会，让群众生活年年有新变化，日子越过越舒心。

　　三、全面加强政府自身建设

　　新形势新任务提出了新的更高要求。我们必须坚持讲政治、尊法治、强担责、尚廉洁，不断强化自身建设，持续改进工作作风，以久久为功的韧性和抓铁有痕的狠劲，切实抓好各项工作落实，全力建设人民满意政府。

　　（一）狠抓政治建设，全面从严治党。

　　贯彻新时代党的建设总要求，全面深入学习贯彻习近平总书记对广东重要讲话和对广东工作一系列重要指示精神，扎实开展“不忘初心、牢记使命”主题教育，树牢“四个意识”，坚定“四个自信”，坚决做到“两个维护”。严明政治纪律和政治规矩，严肃党内政治生活，涵养政府系统风清气正的政治生态。压实意识形态工作责任制，全方位加强阵地建设管理。

　　（二）严格依法行政，建设法治政府。

　　加强重点领域政府立法和重大决策风险评估，推进行政决策科学化、民主化、法治化。健全行政执法体制机制，严格规范公正文明执法。自觉接受市人大和政协监督，认真执行市人大及其常委会决议决定，坚持重大事项向市人大及其常委会报告、向市政协通报，建立实施国有资产管理情况报告制度，高质量办好建议提案。深化政务公开，加强信息发布和政策解读，及时回应社会关切。

　　（三）强化担当实干，建设高效政府。

　　坚决贯彻市委决策部署，实施工作目标倒逼管理，强化督促检查和效能问责，严厉查处懒政庸政怠政行为，以钉钉子的精神雷厉风行抓落实。按照“五个过硬”要求，加强高素质专业化干部队伍建设，提高适应新时代、实现新目标、落实新部署的能力。健全和完善正向激励和容错纠错机制，保护和激发干部干事创业的热情，让只争朝夕、真抓实干、争先创优蔚然成风。

　　（四）加强反腐倡廉，建设廉洁政府。

　　强化政府系统党风廉政建设，履行“一岗双责”。把握运用监督执纪“四种形态”，让党员干部知敬畏、存戒惧、守底线。从严从实整治隐形变异“四风”问题，狠抓工程建设、扶贫惠农、国资国企等领域反腐工作，以坚如磐石的决心正风肃纪。抓好中央巡视整改工作，建立完善相关制度。倡导勤俭节约，建设节约型机关。

　　各位代表！征程万里风正劲，重任千钧再奋蹄。让我们更加紧密地团结在以习近平同志为核心的党中央周围，在省委、省政府和市委的坚强领导下，不忘初心、牢记使命，锐意进取、埋头苦干，干出新作为，不负新时代，努力把阳江打造成沿海经济带的重要战略支点、宜居宜业宜游的现代化滨海城市，为我省实现“四个走在全国前列”、当好“两个重要窗口”作出新的更大贡献，以优异成绩庆祝中华人民共和国成立70周年！
